# More Rescue Stations Urgently Required

**Published:** 1918-08-03

**Authors:** 


### LIFE-SAVING ON THE THAMES.

### More Rescue Stations Urgently Required.

DR. WALDO, the City Coroner, held an inquiry last week concerning the death of William Edward Bolton, aged five, who, with his younger brother, was drowned in the Thames while "fishing" from the Pimlico Stairs. The children lived with their mother, the widow of a soldier killed at the Dardanelles, in Glamorgan Street, Pimlico. A Boy Scout named Murphy, aged thirteen, jumped in and tried to reach the baby, and was well-nigh exhausted when Miss Jeniver Nance, a young lady employed under the Ministry of Pensions, entered the water and rescued him. At the same time her friend, Miss Irene Tisdall, had jumped in, also fully dressed, and tried to rescue the baby. She had just reached him when she found herself at the bottom of the river, and with difficulty just managed to reach the steps.

The Coroner said Miss Tisdall was the sister of Lieutenant Tisdall, V.C., the young Cambridge poet, who dived from the *River Clyde* during the landing on "V" Beach, Gallipoli, and rescued many wounded from the shore under most violent and accurate fire. He was killed a few days later, and the V.C. was granted posthumously. Heroism was evidently one of the characteristics of the Tisdall family. Both ladies and Murphy had risked their lives in the most courageous and plucky manner possible, and were deserving of the highest praise. They had it in evidence that the younger brother was rescued from the river, cold and apparently lifeless, by the Thames Police, after a few minutes' submersion. Also that he had been treated by the police in their boat, and later by a doctor for some twenty minutes, by the Schäfer method of artificial respiration, unfortunately without result.

The Thames Police agree with the Coroner as to the great utility of their station at Waterloo Bridge, the only place of succour on the Thames for the recovery of those apparently drowned. This station, however, was too far away from the Pimlico Stairs to be of any service in the present case. The site of the station had been chosen owing to the number of suicides from the bridge, and a bed with blankets, hot-water baths and bottles, and other remedies were kept there for immediate use. On the Seine, at Paris, numerous stations of this sort were provided, and there, with prompt treatment and the means of applying warmth, especially in cold weather, it was considered a disgrace if people, rescued after five minutes' submersion, were not brought back to life. The City Inspector had told them that in lieu of succour stations at London, Blackfriars, and Tower Bridges, where so many were drowned, reliance was placed by the police on the electric ambulances in which first-aid was administered, while at the same time the patient was quickly conveyed to the nearest hospital for expert treatment. The Coroner also remarked on the valuable teaching in swimming, diving, and life-saving given both boys and girls at the L.C.C. schools. At one of these Murphy had acquired this art.

While they had been told that a man had turned his back on the drowning children and crossed to the other side of the road, Murphy, on the other hand, though not a strong swimmer and fully dressed, had without hesitation gone to their rescue, like the Good Samaritan he was. The Coroner had seen it stated that in the records of the Royal Humane Society for 100 years only two cases could be found of champion swimmers receiving awards for life-saving. In other words, it was not necessary for the rescuer to be a stronger person than the rescued nor an expert swimmer. If properly taught, the danger of loss of life to the rescuer was comparatively slight, though at the same time, as the River Police had told them, currents and other forces were especially deadly in the case of the Thames.

The jury returned a verdict of "Accidental death," and recommended that (*a*) the heroic conduct of Miss Tisdall, Miss Nance, and Boy Scout Albert Murphy be brought to the notice of the Royal Humane Society and the Carnegie Heroes Fund, and (*b*) that the suggestion of the Coroner as to the need of more stations or places of succour on the Thames, for the treatment of those apparently drowned, be brought to the notice of the responsible authority.

